# Letter to the Editor concerning the article “Clinical outcomes of chondroblastoma treated using synthetic bone substitute: risk factors for developing radiographic joint degeneration”

**DOI:** 10.1186/s12957-021-02235-0

**Published:** 2021-06-12

**Authors:** Bo-Wen Zheng, Bo-Yv Zheng, Hua-Qing Niu, Xiao-Bin Wang, Jing Li

**Affiliations:** 1 Department of Spine Surgery, The Second Xiangya Hospital, Central South University, 139 Renminzhong Road, Changsha, 410011 Hunan China; 2 Department of Orthopedics Surgery, General Hospital of the Central Theater Command, Wuhan, 430061 China

## Abstract

The purpose of this letter to the Editor is to report some shortcomings in the statistical analysis and variable grouping in the recent publication of the article “Clinical outcomes of chondroblastoma treated using synthetic bone substitute: risk factors for developing radiographic joint degeneration,” and to further explore some of the factors that may affect the clinical prognosis of chondroblastoma patients. We also suggest future prospective controlled studies with large samples to improve the limitations encountered by Outani et al. (World J Surg Oncol. 18(1):47, 2020) due to insufficient statistical power of variables and lack of controls.

Dear Editor,

We read with interest the recent article by Outani et al. [1]. The authors studied the analysis of 40 patients in a retrospective cohort study. They found that curettage and synthetic bone substitute (SBS) filling can be safely used in the treatment of chondroblastoma (CB) and that postoperative imaging regression of the talus and proximal humerus is more common. We commend the authors for this interesting study because these results contribute to our further understanding of the clinical features and prognosis of CB. However, we still have some questions and suggestions that we would like to share with the authors.

First, we note that the authors performed statistical analyses without multivariate adjustment, which may have exaggerated the significance of some factors, so that in this case, even very strong influencing factors would not be significant. Also, other factors that influence clinical outcomes in CB patients can bias the results. For example, in the study of recurrence, the authors did not consider that there may be different biological behaviors and clinical manifestations of CB at different sites, as indicated by reports that tumors located in the pelvis and proximal humerus are prone to recurrence after surgery [2, 3], and that spinal CB has a higher recurrence rate compared with CB occurring in long bones [4, 5].

Second, the authors included only patients who underwent curettage surgery and did not compare them with patients who underwent other types of surgery, nor did they categorize and compare postoperative adjuvant therapies. This makes the prognostic factors and risk factors appear unclear. A large body of literature reports that different surgical approaches have an important impact on postoperative recurrence [5, 6], and even for cranial CB, surgery is simply the only treatment modality [6]. In a study of CB of the spine, the recurrence rate was 100% even for marginally tumor-free curettage, while patients undergoing total en bloc spondylectomy had no recurrence during follow-up [5]. In patients with CB of long bones, complete scraping of the tumor tissue with bone grafting and bone cement filling or radiofrequency ablation can result in good long-term local control, low recurrence rate, and excellent function [7]. However, the authors did not discuss the study by grouping patients according to the surgical and postoperative adjuvant treatment modalities, ignoring the impact of surgical resection modality and postoperative adjuvant treatment on patients, so whether there is a statistical correlation between patient prognosis and SBS filling after curettage, future comparative studies with large samples are needed to prove this conclusion.

Finally, when the authors performed a univariate Kaplan-Meier curve by log-rank test, they divided the age into high and low groups using 14 years as the cutoff point, which may make some prognostic factors inaccurate. Previous studies have found that the age of prevalence varies in different sites of CB, and the prognosis of patients differs between different age groups, for example, the mean age of patients with cranial and spinal CB is greater than that of patients with long-bone CB [5, 6]; among patients with non-long-bone CB, the prognosis of older patients is significantly better than that of patients with younger CB age [8], so we suggest that the authors use the X-tile software for determining the threshold value of LRFS, i.e., the point corresponding to the minimum P value of the corrected log-rank test [9], a value that can provide valuable guidance for clinical treatment and also help in the clinical management of patients in different age groups.

In conclusion, due to the complex clinical features and biological behavior of CB, its risk and prognostic factors are currently not very clear, and it is particularly important to seek clearer clinical characteristics and prognostic factors. We believe that well-designed prospective studies with comparative analysis of large sample data will help further understanding of CB and even guide treatment and risk avoidance.

## Data Availability

Not applicable.

## Abbreviations

CB: Chondroblastoma
SBS: Synthetic bone substitute
